# Highly stabilized flexible transparent capacitive photodetector based on silver nanowire/graphene hybrid electrodes

**DOI:** 10.1038/s41598-021-88730-6

**Published:** 2021-05-18

**Authors:** Yooji Hwang, Young Hyun Hwang, Kwang Wook Choi, Seungwon Lee, Soojin Kim, Soo Jong Park, Byeong-Kwon Ju

**Affiliations:** Display and Nanosystem Laboratory, School of Electrical Engineering, Korea University, 145, Anam-ro, Seoul, 02841 Republic of Korea

**Keywords:** Electronic properties and devices, Mechanical and structural properties and devices, Optical properties and devices, Graphene, Nanoscience and technology, Nanoscale materials, Nanowires, Optics and photonics, Optical sensors, Electronic devices, Sensors and biosensors, Experimental particle physics, Materials science, Polymers

## Abstract

The need for photodetectors in various fields has gradually emerged, and several studies in this area are therefore being conducted. For photodetectors to be used in various environments, their transparency, flexibility, and durability must be ensured. However, the development of flexible photodetectors based on the current measurement techniques of conventional photodetectors has been difficult owing to the limitations of semiconductor materials. In this study, a new type of flexible and transparent capacitive photodetector was fabricated to address the shortcomings of conventional photodetectors. In addition, by introducing graphene electrodes to a new type of manufactured photodetector, devices with excellent overall chemical, thermal, and mechanical durability have been developed. Compared to photodetectors based on pristine Ag nanowire (AgNW) electrodes, AgNW/graphene hybrid electrode-based photodetectors exhibit a 20% higher photosensitivity. Also, the hybrid AgNW/graphene electrode on the dielectric layer exhibited low sheet resistance (~ 8 Ω/sq) and relatively high transmittance (~ 45%).

## Introduction

A photodetector operates through a mechanism that converts photons into measurable electrical signals. Photodetectors are employed in daily applications such as optical telecommunications, imaging, and biomedical sensing^1,2^. For wide applicability, a photodetector must have good durability in various environments. For example, mechanical durability is fundamental, and the properties of the photodetector must be maintained even in extreme environments such as under high temperature or chemical penetration. To realize flexible and durable photodetectors and other electronic devices, the electrodes that make up the device must also be flexible and durable. In particular, in the case of the photodetector, as mentioned above, the durability of the electrode is of paramount importance because it is a device that converts applied light into an electrical signal and measures it.

In this regard, various flexible and conductive electrodes for photodetectors have been studied, such as carbon nanotubes (CNTs)^3^, graphene^4–8^, metal nanowires^9,10^, metal nanogrids^11,12^, and thin films^13^. Among these alternatives, one-dimensional (1D) metal networks, such as metal nanowires and metal fiber electrodes, have been extensively studied. Numerous studies on metal networks have been conducted, and they have been actively applied owing to their low sheet resistance (10 Ω/sq), high optical transmittance (approximately 90%), and high strain value (approximately 11%)^14^. In particular, among these 1D metal network electrodes, AgNWs do not require a high vacuum or high-temperature deposition process and can be developed via roll-to-roll manufacturing^15^. For this reason, studies using AgNWs are being actively conducted.

However, AgNWs have fatal disadvantages as electrodes, such as low adhesiveness, high surface roughness, and low durability, a common disadvantage of metal electrodes^16–18^. Typical AgNWs exhibit a diameter of ~ 40 nm, a length of ~ 120 µm, and an aspect ratio of up to 2500^19^. Because AgNWs have an extremely small length, a large amount of AgNWs must be coated to exhibit sufficient electrical characteristics, that is, to achieve a low percolation threshold. As more AgNWs are coated, the electrical properties are improved; however, the transmittance decreases. As a result, AgNWs do not have sufficient properties to be used as a transparent electrode^20^. In addition, because AgNWs are manufactured based on a spin-coating process, high surface roughness is exhibited at the junction where the nanowires overlap. Finally, to solve the structural problems of AgNW, the fabrication of a gas- and thermal-stable, conductive, and flexible buffer layer for the AgNW electrodes is required, which can improve the non-uniform current flow caused by the network structure while maintaining the durability of the AgNW electrodes.

Graphene represents a two-dimensional (2D) graphite structure and is considered the most promising material with outstanding electrical, optical, and thermal properties because it can prevent the penetration of small molecules thanks to its unique honeycomb structure^21,22^. Based on a previous study^23^, graphene was transferred to the top surface of the AgNW electrodes to improve the chemical and thermal durability while fabricating an AgNW-based photodetector.

In this study, the fabricated photodetectors are capacitance-based photodetectors rather than conventional ones representing a current measurement base. Conventional photodetectors are based on the principle that an electron–hole pair is generated when photons of the same or higher energy are received in an active layer made of a semiconductor having a specific bandgap^24–28^. At this time, a photocurrent is produced as electrons move to the conduction band and holes to the valence band. A photodetector employing such a current measurement method is difficult to develop into a flexible and highly durable device. Most highly efficient thin-film semiconductor materials are highly brittle; hence, it is extremely difficult to fabricate them using flexible photodetectors. Through neutral plane engineering, slightly flexible photodetectors have been developed; however, the process is somewhat difficult and inefficient.

In our previous study, a new type of capacitive photodetector was introduced that didn’t exist before^29–31^. The fabricated device is based on a structure wherein ZnS:Cu particles are dispersed in a polymer film and transparent electrodes, which are AgNWs formed on both surfaces of a composite film. By dispersing the particulate semiconductors into a flexible polymer rather than forming a thin film, the problem of easily breaking semiconductor-based photodetectors has been solved. In addition, inspired by the fact that the dielectric properties of composite films, including semiconductor particles, change upon light irradiation, it is possible to measure the capacitance generated in the composite film by using surface electrodes. In this study, for the transparent electrodes formed on both surfaces of the film, AgNWs and AgNW/graphene electrodes are used. Owing to the AgNW/graphene electrode used on the top surface of the film, the overall durability of the photodetector was further improved. A new type of next-generation flexible and durable photodetectors can be realized by introducing a graphene electrode to a capacitive photodetector whose manufacturing process is extremely simple and measurement method and principle are intuitive.

## Experiment

### Preparation of ZnS:Cu-PVB (polyvinyl butyral) solution

To fabricate the PVB solution, *N*,*N*-dimethylformamide (DMF, Sigma-Aldrich Chemicals, USA), Butvar (B-98, M_n_ $\approx$ 36,000 g/mol), and hexamethylene diisocyanate (HMD, Daejung Chemicals & Metals, Rep. of Korea) were mixed at a weight ratio of 10:4:0.6. The solution was stirred for 12 h at 200 rpm. After the solution became clear, ZnS:Cu particles (copper content of 9 at% and an average diameter of $\approx$ 20 µm, National EL Technology, Rep. of Korea) were dispersed in the solution at ratios of 1:0.3 (23 wt%), 1:0.5 (33 wt%), 1:0.7 (41 wt%), and 1:0.9 (47 wt%).

### Fabrication of AgNW/graphene-based capacitive photodetector

A glass (Eagle XG, Corning Co., Ltd., USA) was immersed in acetone, methanol, and deionized (DI) water and washed for 10 min each time through ultrasonication. In addition, 1.0 wt% of AgNWs (average diameter and length of 25 nm and 30 µm, respectively, Duksan Hi-Metal Co., Ltd., Rep. of Korea) in isopropanol was spin-coated on the glass at 500 rpm for 30 s and dried for 1 min at 100 °C. The prepared ZnS:Cu-PVB solution was then spin-coated on the AgNW-coated glass at 500 rpm and baked for more than 2 h at 85 °C. For the top electrode of the photodetector, AgNWs were spin coated onto the glass after the PVB solution was baked. To safely peel them off the preliminary glass substrate, the prepared samples were then soaked in DI water for 2 h to induce hygroscopic swelling of the composite film. Finally, to ensure the durability of the manufactured photodetector, graphene was transferred onto the composite film. The graphene monolayer was transferred using a poly (methyl methacrylate) (PMMA)-based wet-transfer technique (PMMA, MicroChem 950 C4, 4% in chlorobenzene, molecular weight of 950,000). The detailed fabrication process is described in our previous paper^23^.

### Measurement

A four-probe system comprising equal tip spacing was used to measure the sheet resistance over randomly selected points of each sample and estimate the electrical properties of the AgNW/graphene hybrid electrodes. AgNW/graphene hybrid electrodes based a capacitive photodetector were analyzed using a field emission scanning electron microscope (SEM; S-4800, Hitachi, Ltd.), and the optical properties were measured using a Cary 5000 UV–visible spectrophotometer (Varian/Agilent). A cyclic bending test was conducted using a bending tester (z-tec, Inc.) with a digital multimeter to determine the real-time line resistance. To investigate the chemical stability, pH solutions were prepared by mixing a solution of hydrochloric acid (HCl) and sodium hydroxide (NaOH) with deionized water^23^. An inductance–capacitance–resistance (LCR) meter (HP4284A, Agilent) was used to measure the sample capacitance upon light irradiation^31^.

## Results and discussion

### Theoretical background of capacitive photodetector

The increase in the dielectric permittivity of the semiconductor with particle-polymer composites upon irradiation of light with a specific wavelength changes the capacitance of the composite sandwiched between the two facing electrodes. Two physical theories of “interfacial polarization” and the “Maxwell–Wagner-Sillars (MWS) effect” at particle–polymer interfaces can explain these changes in the dielectric response of the composites upon light irradiation^32–34^. The MWS effect is known to occur in the heterojunction structures owing to the accumulation of charges at the interfaces as well as the formation of dipoles on particles or clusters. In the heterostructures, additional charge accumulations occur at the particle boundaries under an external electric field owing to the differences in dielectric characteristics (ability to hold charges) and electrical conductivity^35^. It is well known that the huge difference in the conductivity of the particles and polymers will lead to a large charge accumulation at the interface because the current cannot flow freely across it. The accumulated charges are directly proportional to the difference between the two conductivities, and the interface behaves like a nanocapacitor^33^. Thus, the assembled movements of the interfacial dipoles at the interfaces amplify the response to the incoming electromagnetic field and thus enhance the microwave absorbing capability. Based on these theories, in this study, a composite film was fabricated by dispersing ZnS:Cu particles in polyvinyl butyral (PVB), a flexible free-standing polymer. The fabricated devices were mechanically flexible and able to withstand bending with a curvature radius of several millimeters.

### Fabrication of AgNW/graphene capacitive photodetector

**Figure 1:**
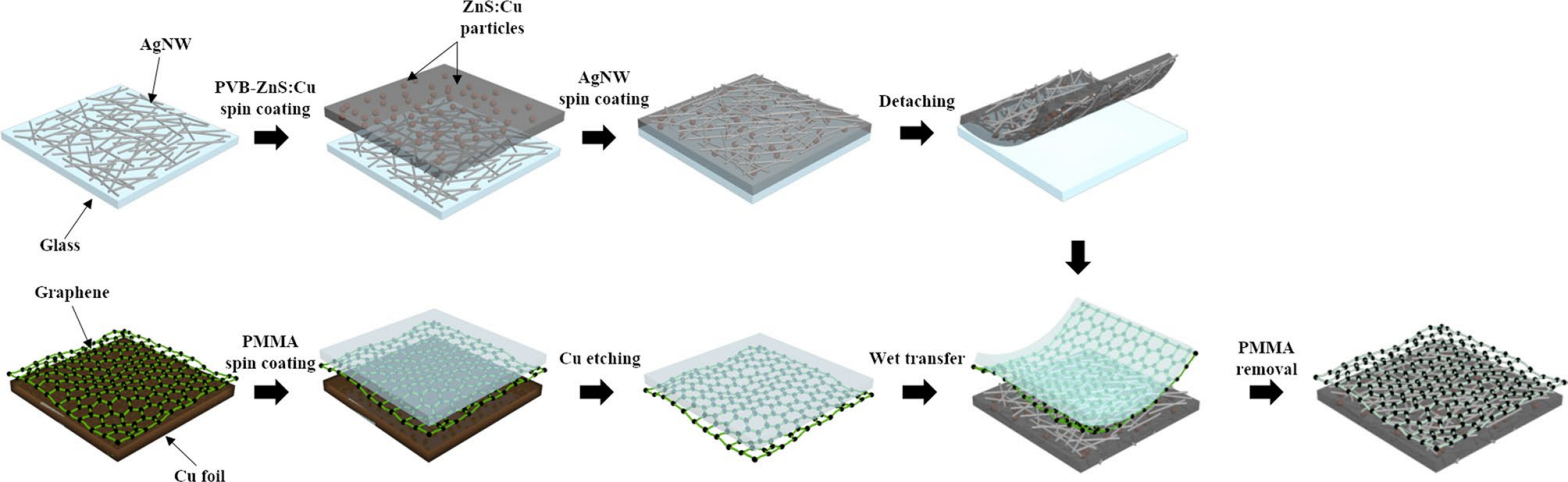
Fabrication process of the AgNW/graphene hybrid electrode-based capacitive photodetector.

Figure 1 shows a schematic of the fabrication process for the AgNW/graphene-based capacitive photodetector. As can be seen in Fig. 1, the AgNW electrodes for the bottom surface of the composite film are embedded and are stable under external stimuli, whereas the AgNW electrodes on the top surface are vulnerable to the external environment because they are simply spin coated on the top. For this reason, stable bonding with the structure at the bottom is not achieved, and thus may fall off from surface. Therefore, the CVD-grown graphene monolayer was wet-transferred to the top of the capacitive photodetector. By transferring the graphene to the top, it is possible to increase the thermal and chemical durability of the entire device as well as the top AgNW electrodes. In addition, graphene has high flexibility; therefore, it can be advantage in the characteristics of the manufactured flexible capacitive photodetector.

**Figure 2:**
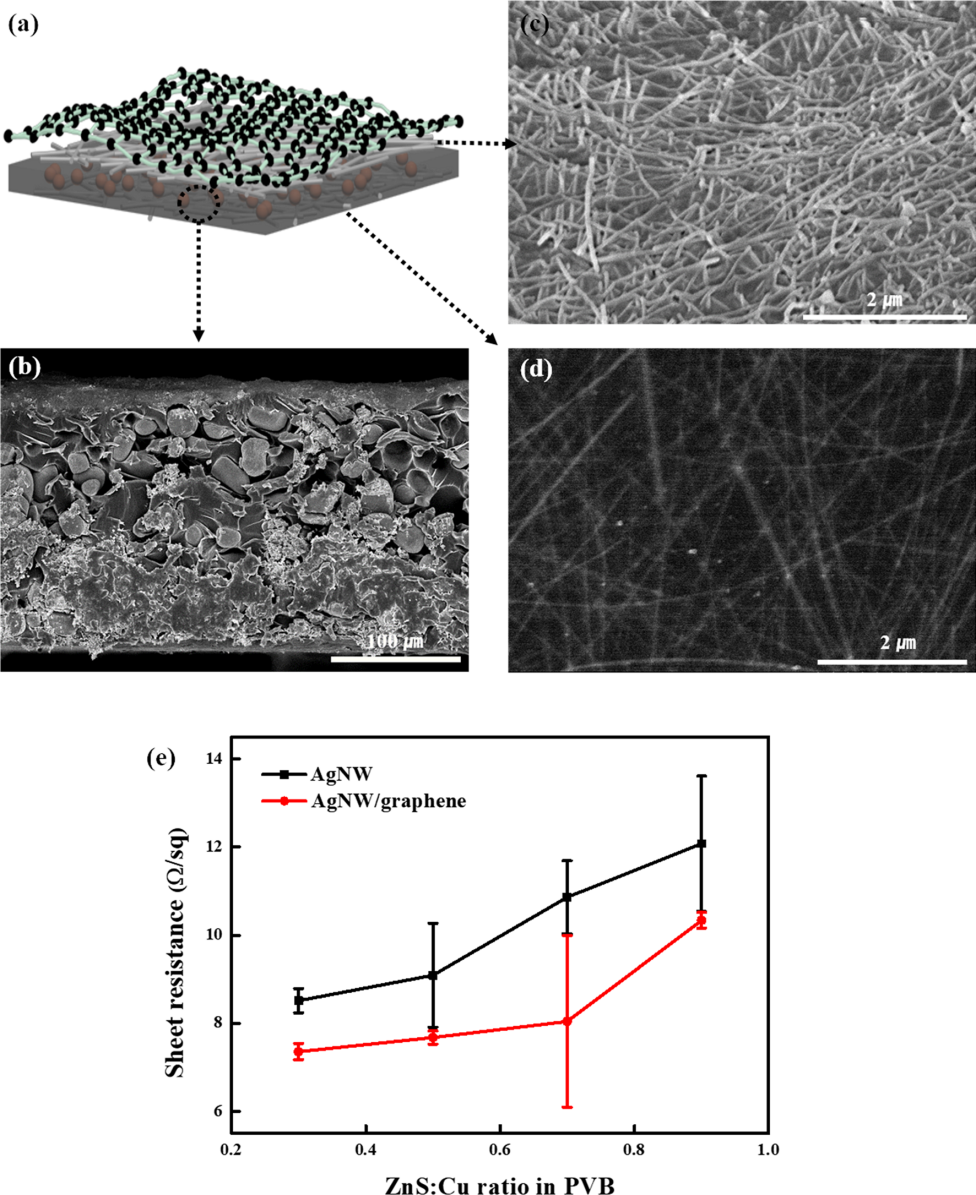
(**a**) Schematic description of the fabricated capacitive photodetector, (**b**) SEM image of cross-section of the composite layer composed of ZnS:Cu-PVB, (**c**) AgNWs deposited on top of the composite layer, (**d**) AgNWs embedded at the bottom of the composite layer, and (**e**) sheet resistance of AgNW and AgNW/graphene hybrid electrode-based capacitive photodetector.

Figure 2 shows SEM images of a cross-section of the photodetector as well as the upper and lower AgNW electrodes. ZnS/Cu particles with an average diameter of ~ 20 µm were dispersed in polyvinyl butyral (PVB), a flexible free-standing polymer, as shown in Fig. 2a,b. Because the particles are uniformly dispersed in flexible polymers, the fabricated photodetector is mechanically flexible and able to withstand bending with a curvature radius of several millimeters. Here, because the specific surface area of the spherical ZnS/Cu particles was much smaller than those of most nanosized particles (nanoparticles), aggregation between the particles was not observed. Figure 2c,d show the top and bottom AgNW electrodes, respectively. The morphologies of the AgNWs are quite different: The top AgNWs are simply spin-coated on top of the ZnS:Cu-PVB, whereas the bottom AgNWs are embedded into ZnS:Cu-PVB. Therefore, the bottom AgNWs featured a significantly flattened shape, and the SEM image of the bottom AgNWs seemed to be slightly blurred. By contrast, the top AgNWs coated on the ZnS:Cu-PVB surface did not appear to be firmly adhered; therefore, the graphene buffer layer was wet-transferred to the top of the AgNWs.

By transferring graphene to the top of the AgNWs, adhesion of the upper AgNWs can be increased, and an additional current path can be formed. The average sheet resistances of the AgNW electrodes obtained at the ZnS:Cu ratios of 0.3, 0.5, 0.7, and 0.9 wt% in PVB were 8.516, 9.09, 10.864, and 12.076 Ω/sq, respectively, as shown in Fig. 2e. After the introduction of graphene, the average sheet resistances were 7.356, 7.676, 8.042, and 10.34 Ω/sq, respectively, which are approximately 25% lower than those of the AgNW electrodes.

Here, for both AgNW electrodes and AgNW/graphene hybrid electrodes, the sheet resistance of the substrate increased as the ratio of ZnS:Cu particles in PVB increased. As the number of particles increases, the density of particles inside the thin polymer layer increases. This increases the roughness of the polymer layer, resulting in an increase in resistance.

Compared to those of the AgNW electrodes, the sheet resistance of the AgNW/graphene hybrid electrodes was lower, which suggests that the AgNW/graphene hybrid electrodes delivered a better electrical performance than the AgNW electrodes. This is because, as the AgNW electrodes feature a network form, there are void spaces where the electrodes are not present, which eventually leads to an open-circuit fault. In addition, AgNWs are known to have a large surface roughness, which can easily penetrate the thin film layers. For the AgNW/graphene hybrid electrodes, the CVD graphene layer covered the entire AgNW electrodes, and the current flowed throughout all non-empty spaces. In addition, covering the CVD graphene layer has the advantage of solving the problem of the large surface roughness of the AgNWs.

### Optical characteristics of AgNW/graphene capacitive photodetector

**Figure 3:**
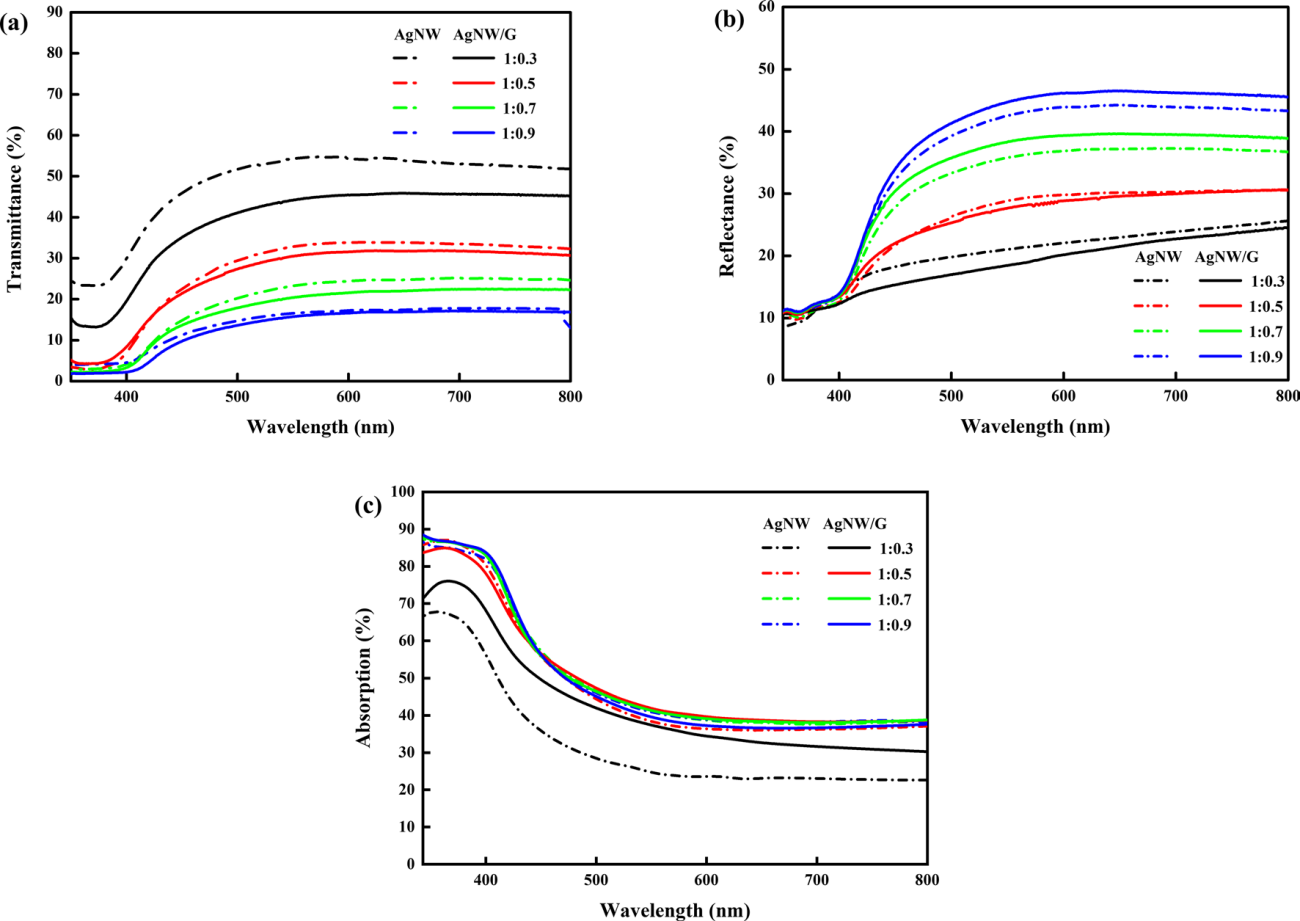
(**a**) Total transmittance, (**b**) reflectance and (**c**) absorption of the AgNW and AgNW/graphene electrode-based photodetectors obtained using different ZnS:Cu particle ratios in PVB.

To investigate the optical properties of the AgNW/graphene capacitive photodetector, the transmittance, reflectance and absorption were measured depending on the ZnS:Cu particle ratio in PVB (Fig. 3a–c). Overall, the transmittance decreases as the ZnS:Cu particle ratio increases because the embedded density of the particles also increases. Thus, devices with a ZnS:Cu loading ratio of 0.3 featured a high transmittance with a low reflectance over the entire visible range. For the AgNW electrode-based photodetector, the maximum transmittances obtained at ZnS:Cu ratios of 0.3, 0.5, 0.7, and 0.9 wt% were 54.8%, 34.0%, 25.3%, and 17.8%, respectively. For the AgNW/graphene hybrid electrode-based photodetector, the maximum transmittances were 45.9%, 31.8%, 22.5%, and 17.2%, respectively. On average, the photodetectors using the AgNW/graphene hybrid electrode exhibit a 4% lower transmittance than the photodetectors using the AgNW electrodes. It can be inferred that this is based on the light absorption rate of graphene, which is known to be 2.3% on average.

The measured transmittance of the photodetector can be evaluated as relatively low, and this is because the photodetectors were manufactured using 1.0 wt% of AgNWs. As the concentration of AgNWs increases, the overall device transmittance decreases, and the sensitivity decreases^29^. From this perspective, the AgNWs concentration should be lowered to increase the transmittance and sensitivity; however, in this study, the device was fabricated using a 1.0 wt% dispersion, which represents a relatively high density of AgNWs, to enhance the flexibility of the device. It is well known that the resistance of the AgNW electrodes is increased via repeated bending owing to the decreased number of conduction paths made by AgNWs^33^. When a photodetector is manufactured with low-concentration-based AgNWs, the resistance increases rapidly upon mechanical stress, and an excessively large increase in resistance hinders the transfer of charges from the power source to the dielectric, which can cause the capacitance to decrease. In conclusion, photodetectors fabricated with a 1.0 wt% AgNWs dispersion with graphene exhibited a transmittance of approximately 45% of incident light with a 550-nm wavelength.

In addition, we can see distinctive aspects depicted in Fig. 3a,b. The transmittance of AgNW electrodes is higher than that of AgNW/graphene about all ZnS:Cu ratios in Fig. 3a. However, as shown in Fig. 3b, the change of reflectance according to the presence of graphene is independent of the ZnS:Cu ratios. This can be explained by relative properties difference between AgNW and AgNW/graphene. In Fig. 3b, reflectance spectra are affected by light scattering. This can explain the reflectance difference between AgNW and AgNW/graphene at the same ZnS:Cu ratio. In the cases of 1:0.7 and 1:0.9 ratios, the presence of graphene clearly helps reduce the light scattering whereas in the cases of 1:0.3 and 1:0.5, the graphene does not seem to reduce light scattering but even increase it.

The absorption spectra of different ZnS:Cu particle ratios are depicted in Fig. 3c to show photoresponse characteristics via absorption spectra. The dotted and solid lines clearly exhibit the effect of graphene layer on AgNW. Regardless of the ZnS:Cu content, the bandgap of the ZnS:Cu-PVB composite is known to be 2.95 eV. This explains to an increase of photons absorption below 423 nm of wavelength. The bandgap energy was calculated based on the Tauc plot equation, the details of which are described in our previous study^30,31^.

### Photoswitching characteristics of AgNW/graphene capacitive photodetector

**Figure 4:**
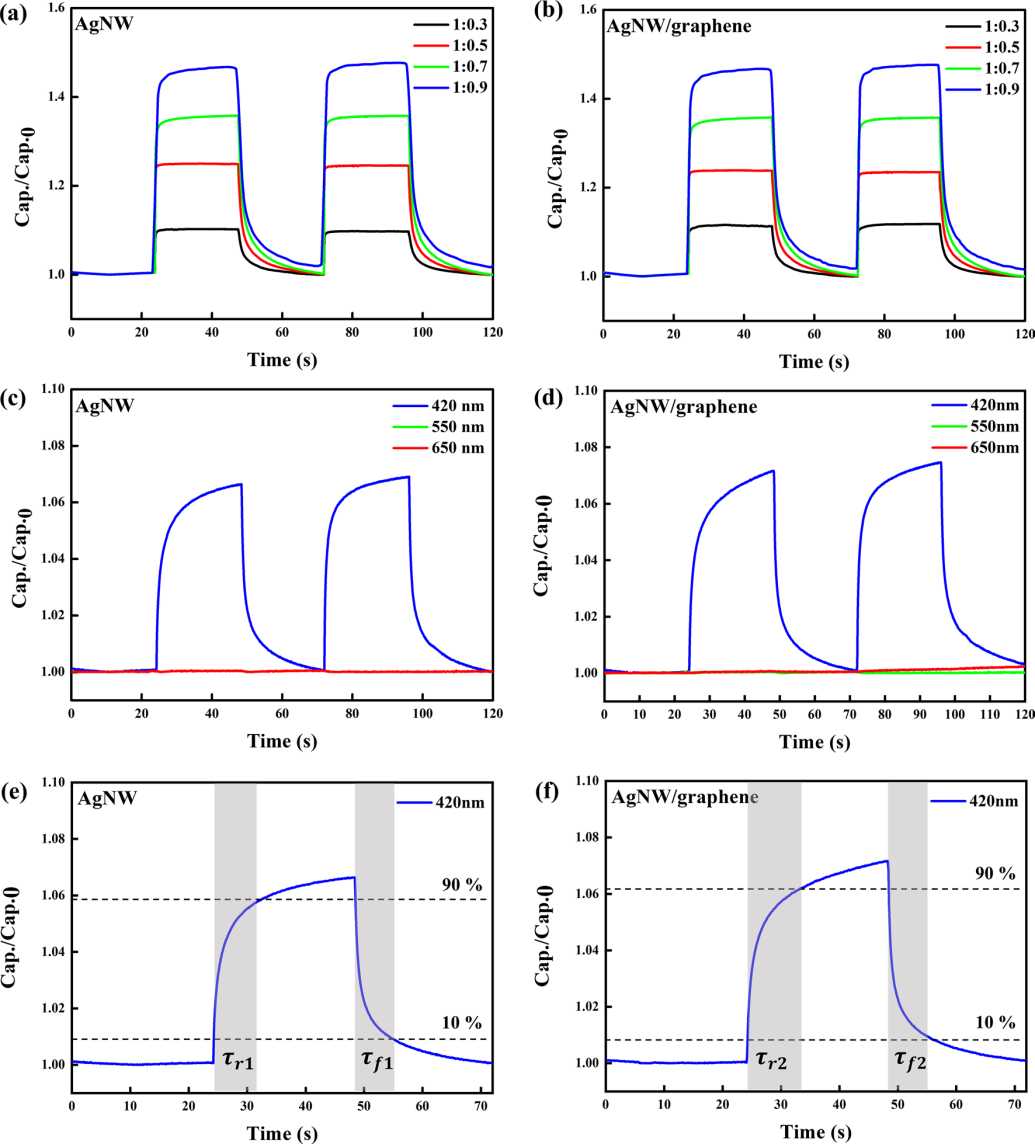
Photoswitching characteristics of capacitive photodetectors (**a**) upon light on–off (420 nm, 1.2 mW/cm^2^) irradiation cycling determined for various ZnS:Cu ratios in PVB based on AgNW electrodes, (**b**) AgNW/graphene electrodes, and (**c**) under light on–off (1.2 mW/cm^2^) irradiation of three different wavelengths (420, 550, 650 nm) based on AgNW and (**d**) AgNW/graphene, and (**e**) response and recovery time of AgNW and (**f**) AgNW/graphene.

Based on the calculated ZnS:Cu-PVB composite bandgap energy, a lighting source with a wavelength of 420 nm and a power of 1.2 mW/cm^2^ was employed to test the photoresponsivity of the photodetectors. The photoresponsive capacitance was measured under ambient conditions using a two-probe method and a 50-kHz signal. The ZnS:Cu particle ratio-dependent photoresponsivities of the AgNW-based photodetector and that of the AgNW/graphene-based photodetector are shown in Fig. 4a,b, respectively. For the AgNW-based photodetector, photoresponsivities enhanced by 1.1-, 1.25-, 1.36-, and 1.47-fold are shown compared to those at the off state at ZnS:Cu particle ratios of 0.3, 0.5, 0.7, and 0.9, respectively. Similar to the AgNW/graphene-based photodetector, photoresponsivities enhanced by 1.1-, 1.24-, 1.35-, and 1.47-fold are shown at ratios of 0.3, 0.5, 0.7, and 0.9, respectively. The photoresponsivities revealed that the capacitance of the composite layer showed a higher photosensitivity with a higher particle density, which indicates the existence of a trade-off between the transparency and sensitivity of the fabricated photodetectors.

To measure the photoresponsivities of the fabricated photodetectors, capacitances at different wavelengths were also observed, as shown in Fig. 4c,d. For both AgNWs and AgNW/graphene-based photodetectors, the capacitance did not effectively change upon irradiation by light with a wavelength of 550 or 650 nm, which shows that the implemented photodetectors were highly photosensitive. The photodetector reacted only at a wavelength of 420 nm due to the bandgap energy. In Fig. 4e,f, response time ($\tau_{r}$) is computed to be the time difference between 10 and 90% of Cap./Cap.0 at a positive slope whereas recovery time ($\tau_{f}$) is to be the difference at a negative slope^36^. From Fig. 4c,d, the response times and recovery times for both AgNW ($\tau_{r1}$ and $\tau_{f1}$) and AgNW/graphene ($\tau_{r2}$ and $\tau_{f2}$) based photodetectors are $\tau_{r1} = 9.33 s$, $\tau_{{{\text{f}}1}} = 9.41 s$, $\tau_{r2} = 11.55 s$, $\tau_{f2} = 8.95 s$, respectively.

As mentioned earlier, as the thickness of the electrodes increases, the overall device transmittance decreases, and the sensitivity decreases. This is because as the thickness of the electrode increases, the light transmitted through the ZnS:Cu-PVB composite film decreases, and the amount of charge to be transferred decreases. In the case of the AgNW/graphene photodetector, compared to the AgNW electrode-based photodetector, because the thickness of the electrode becomes thicker owing to the additional graphene electrode, it can be predicted that the photoresponsivity will be relatively lower. In addition, the result indicates that the transmittance of the AgNW/graphene photodetector is approximately 4% lower than that of the AgNW electrode-based photodetector. Nevertheless, it can be seen that the measured photoresponsivity of both photodetectors shows little difference. It can be confirmed that the lowered transmittance, which is a disadvantage caused by the additional use of the graphene electrode, does not significantly affect the photoresponsivity of the photodetectors.

### Durability of AgNW/graphene capacitive photodetector

The fabricated AgNW/graphene-based photodetectors exhibited a superior mechanical flexibility, which is essential for emerging flexible optoelectronic devices. A repetitive bending test was conducted to investigate their mechanical durability. During the test, photodetectors based on AgNW and AgNW/graphene electrodes were rolled at various bending radii (*r*_b_) or rolled and subsequently unrolled at a bending radius of 5 mm at a speed of 3 mm s^−1^. The sheet resistance of each photodetector was compared to its initial value (Δ*R*/*R*_0_, where Δ*R* is the change in resistance after bending, and *R*_0_ is the initial resistance). The tensile strain applied to the bent substrate was estimated using^37^

$$ {\text{Strain}}\left( {\text{\% }} \right) = { }\frac{{d_{substrate} + d_{electrode} }}{{2 \times R_{c} }}, $$

where $d_{substrate}$ and $d_{electrode}$ are the thicknesses of the substrate and electrodes, respectively, and $R_{c}$ is the radius of the curvature.

**Figure 5:**
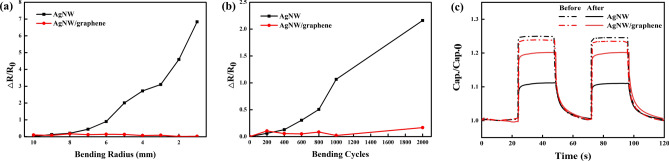
(**a**) Relative difference in resistance as a function of the (**a**) radius of curvature and (**b**) number of bending cycles at a bending radius of 5 mm, and (**c**) photoswitching characteristics before and after 2000 bending cycles test.

In Fig. 5a, the mechanical flexibilities of the photodetectors based on AgNW and AgNW/graphene electrodes are compared at various bending radii (r_b_). The AgNW-based photodetector exhibited changes in resistance of more than 100% at above r_b_ = 6 mm and finally reached 700% of the initial resistance. This is because the polyvinyl pyrrolidone (PVP) on the surface of the nanowires generated during the manufacturing process weakens the adhesion to the substrate^38^, and as the bending radius decreases, the AgNWs at the top of the composite layer fall off. By contrast, there was little change in the resistance of the AgNW/graphene photodetector. Figure 5b compares the mechanical flexibilities of the photodetectors based on AgNWs and AgNW/graphene as functions of the number of wrapping cycles. The resistance of the AgNW/graphene-based photodetector was slightly changed after 1,000 bending cycles, whereas that of the AgNW-based photodetector was increased by more than 100% after 1,000 bending cycles and by 200% after 2,000 bending cycles. As shown in Fig. 5c, the photoswitching characteristics of the photodetectors after the mechanical test were measured. After the bending test, the sensitivity of the AgNW-based photodetector was significantly lower than that of the AgNW/graphene photodetector, despite the photoswitching curve remaining stable. It can be expected that, after the bending test, the AgNWs were detached from the composite film owing to the low adhesion of the AgNWs. When the AgNWs were detached, the void space of the electrode of the network type increased, and thus, the amount of charge accumulated in the composite film decreased, thereby reducing the overall sensitivity. However, in the case of the AgNW/graphene photodetector, the monolayer graphene film enhanced the adhesion of the AgNW electrodes to the composite film, and thus, the sensitivity of the photodetector was high.

**Figure 6:**
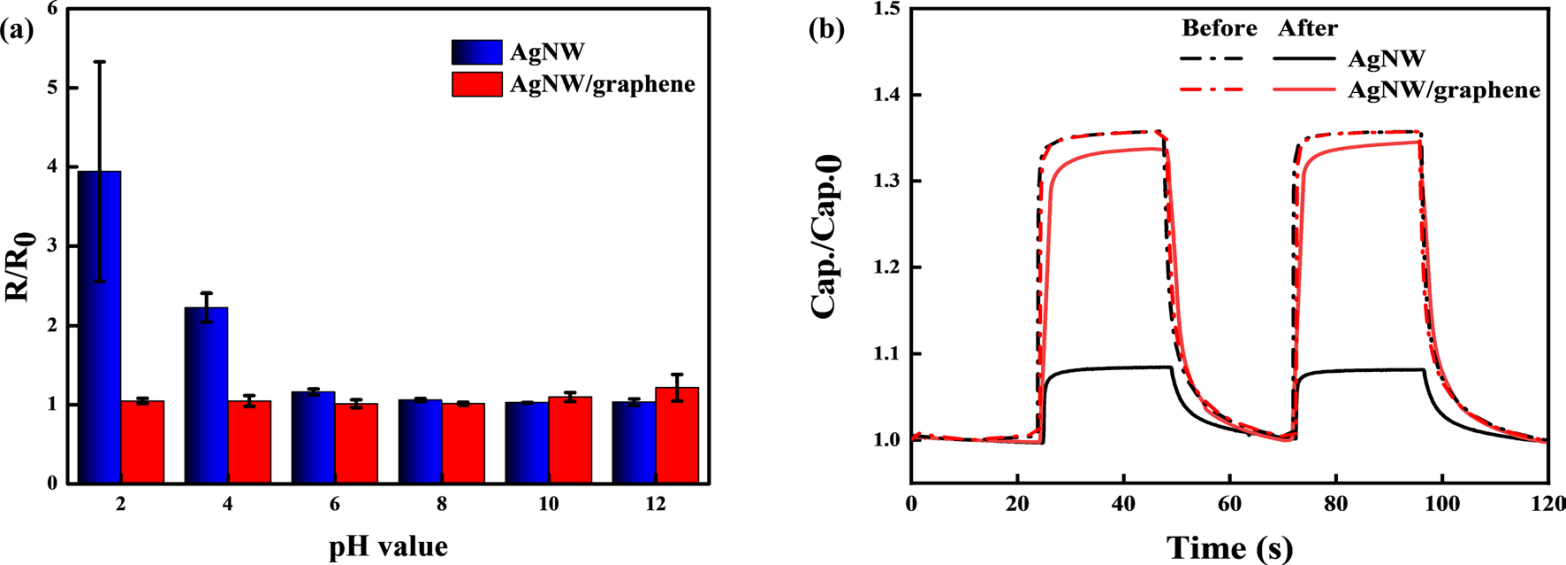
(**a**) Variation in the resistance after immersion in solutions with different pH values and (**b**) photoswitching characteristics before and after pH 2 chemical stability test.

To further evaluate whether the graphene layer acted as a protective layer against the AgNW electrodes, various solutions with pH values ranging from 2 to 12 were prepared. The AgNW and AgNW/graphene electrode-based photodetectors were then covered at each pH for 20 min. As shown in Fig. 6a, the resistances of the AgNW electrode-based photodetector immersed in the solutions with a pH of 6 to 12 were not significantly different, whereas, at a pH of 2 and 4, the changes in resistance were approximately 2.2% and 3.9%, respectively. The resistance of the AgNW/graphene photodetector was not considerably increased over the entire pH range of 2–12. The unique honeycomb lattice of the graphene acts as a protective barrier that prevents the penetration of small molecules; therefore, the AgNWs were able to maintain the properties of the electrode without oxidation. The AgNW/graphene hybrid electrode-based photodetector exhibited chemical stability. Figure 6b shows the photoswitching characteristics of the photodetectors. The significant gap in the change in photosensitivity between the AgNW and AgNW/graphene electrode-based photodetectors also verifies that graphene has high chemical stability, thereby improving the chemical durability of the overall photodetector.

**Figure 7:**
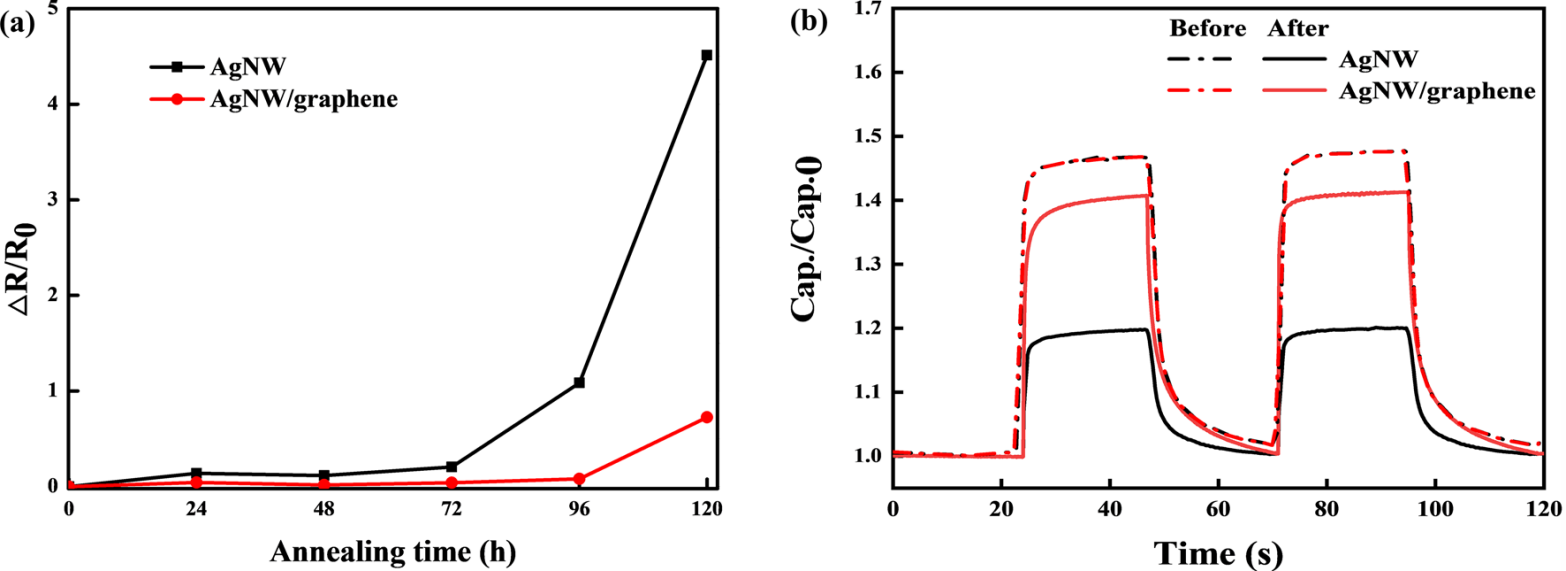
(**a**) Variations in resistance when exposed to a temperature of 160 °C and (**b**) photoswitching before and after the 120 h thermal stability test.

Finally, to further evaluate the thermal stability of the AgNW/graphene-based photodetector through the thermal stability of the graphene protection layer, the photodetectors based on AgNWs and AgNW/graphene were annealed on a hot plate at 160 °C for 120 h. The variations in resistance were measured every 24 h. As shown in Fig. 7a, the resistance of the AgNW electrode-based photodetector rapidly increased after 72 h and then reached a value that was almost 400% higher than the initial resistance. The AgNWs began to melt as high temperatures were applied, and the overlapping parts became welded. However, when a continuous high temperature was applied, dewetting occurred, and the shape of the nanowires was not maintained; they aggregated, forming round silver nanoparticles. Therefore, after the AgNWs melted at 160 °C, the contacts between the AgNWs were initially welded, but after 72 h, the part in contact was cut off, and the overall resistance increased. By contrast, the AgNW/graphene hybrid electrode-based photodetector exhibited a slightly increased resistance, which demonstrates its superior thermal stability to that of the pristine AgNW electrode-based photodetector. Although both photodetectors exhibited a relatively stable photoswitching curve, as shown in Fig. 7b, the AgNW electrode-based photodetector showed a sensitivity decrease of approximately 20% compared to that of the AgNW/graphene hybrid electrode-based photodetector.

Overall, the fabricated AgNW/graphene hybrid electrode-based photodetector exhibited an excellent performance, compared to the AgNW electrode-based photodetector, and presented stable photoswitching responsivity without being affected by the surrounding environment, that is, it was unaffected by chemicals or temperature changes. The overall results suggest that the AgNW/graphene hybrid electrode-based photodetector is expected to be used in various next-generation optoelectronic devices.

## Conclusions

We fabricated a highly stable and flexible AgNW/graphene hybrid electrode-based capacitive photodetector by wet-transferring CVD-grown monolayer graphene onto AgNW electrodes. The fabricated photodetector was inspired by the fact that the dielectric permittivity of a given semiconductor increases upon irradiation by photons with an energy equal to or higher than the semiconductor band-gap energy. The simple structure of the photodetector is composed of embedded AgNWs underneath the composite layer and another layer of AgNWs deposited on the opposite side of the composite layer, and graphene is additionally transferred to increase the adhesion of the AgNWs. Because the CVD graphene layer acts as both an excellent gas-barrier layer and a flexible buffer layer simultaneously, the AgNW/graphene hybrid electrode-based photodetector exhibits excellent chemical, thermal, and mechanical flexibility. We anticipate that this unconventional technology will advance the development of various wearable devices for next-generation electronics.

## Data Availability

All data generated or analysed during this study are included in this published article.
